# Sea Buckthorn in Cardiovascular and Metabolic Health: Evidence for the Role of the Leaves

**DOI:** 10.3390/nu18183009

**Published:** 2026-09-14

**Authors:** Aleksandra Kordas, Michał S. Majewski

**Affiliations:** Department of Pharmacology and Toxicology, Faculty of Medicine, Collegium Medicum, University of Warmia and Mazury in Olsztyn, 10-082 Olsztyn, Poland

**Keywords:** *Hippophae rhamnoides*, polyphenols, flavonol glycosides, ellagitannins, isorhamnetin, endothelial dysfunction, oxidative stress, vascular function, bioavailability, cardiometabolic health

## Abstract

Most research on sea buckthorn (*Hippophae rhamnoides* L.) has focused on the berries and oils. This structured narrative review examines the chemical profile of the leaves and evaluates cardiovascular and metabolic evidence from leaf preparations, isolated constituents, and other sea buckthorn materials. Two complementary PubMed searches yielded 3539 unique records. After screening in successive stages, 85 publications were included. A supplementary search in Scopus yielded no unique records. Leaves contain glycosides of isorhamnetin, quercetin, and kaempferol, together with ellagitannins, proanthocyanidins, phenolic acids, and triterpenoids. Their relative amounts vary between cultivars, harvest, processing, extraction, and the denominator used for reporting. Leaf preparations improved selected metabolic, vascular, or inflammatory outcomes in several animal and ex vivo models. Studies of isolated flavonols support biological plausibility but do not identify the compounds responsible for effects of complete leaf preparation. Human trials have examined berries, purée, juice and oils; effects on lipids, glucose regulation, blood pressure, and platelet activity were generally small or inconsistent. No controlled human trial or human pharmacokinetic study has evaluated a chemically characterized leaf preparation. This absence is the main obstacle to clinical translation. The next stage should compare one defined leaf preparation with appropriate controls in validated animal models, test fractions prepared from the same leaf batch, and require replication before human studies of pharmacokinetics, dose, safety, and efficacy. Current evidence supports this sequence but does not justify therapeutic use of sea buckthorn leaves.

## 1. Introduction

Endothelial dysfunction, oxidative stress, inflammation, and impaired regulation of lipid and glucose metabolism are connected features of cardiometabolic disease. Plant polyphenols can modify these processes, but their effects depend on chemical form, exposure, and the preparation tested [1,2,3]. This background provides mechanistic context and does not establish an effect of sea buckthorn leaves.

**Figure 1:**
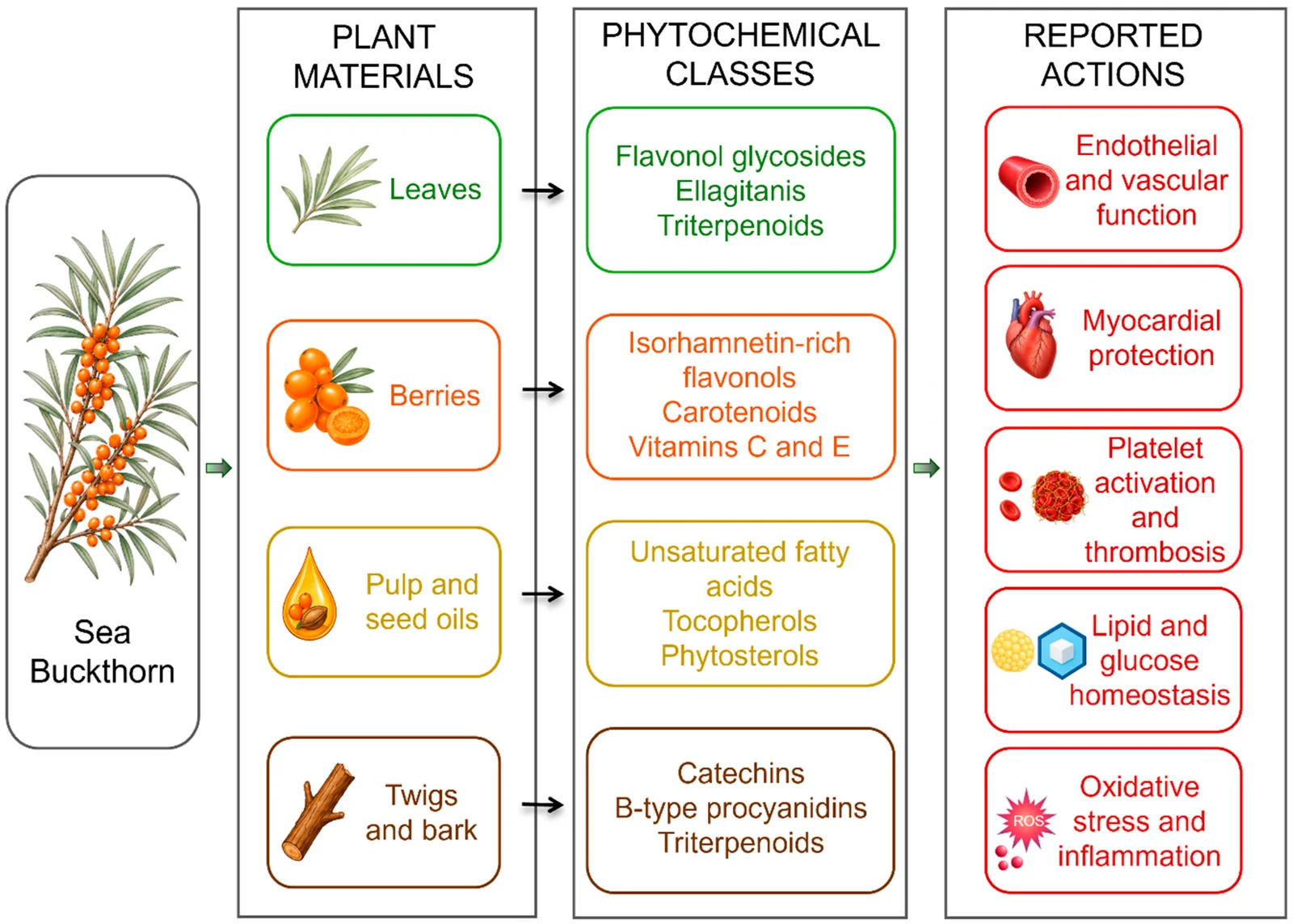
Sea buckthorn plant materials, major phytochemical classes, and reported cardiovascular and metabolic actions. Leaves are particularly rich in flavonol glycosides, ellagitannins, and triterpenoids; berries contain isorhamnetin flavonols, carotenoids, and vitamins C and E; pulp and seed oils provide unsaturated fatty acids, tocopherols, and phytosterols; and twigs and bark contain catechins, B-type procyanidins, and triterpenoids. The reported actions summarize evidence across preparations and isolated constituents and do not indicate that every material has demonstrated efficacy in humans.

Sea buckthorn (*Hippophae rhamnoides* L., SB) occurs across Eurasia as a thorny deciduous shrub or, less commonly, a small tree. In Asia, both the fruit and leaves have traditionally been used in food and medicine. Berries are the main material used in modern sea buckthorn products. Besides vitamins and minerals, they contain carotenoids and a lipid fraction distributed between the seeds and the fruit flesh, from which oils can be obtained [4]. Compared with berries, sea buckthorn leaves have been less widely used and investigated, despite being rich in polyphenols and flavonoids. Sea buckthorn contains numerous flavonoids, of which isorhamnetin and quercetin glycosides form the main groups; kaempferol glycosides are also well represented [5]. Comparisons with berries generally show that leaves contain higher levels of total polyphenols and flavonoids [6]. Analyses of leaves and teas prepared from them have revealed glycosides of all three flavonols, accompanied by hydrolyzable tannins and several phenolic acids. In chemical assays, raw leaves and green leaf tea had greater antioxidant activity than black leaf tea. All three materials produced moderate inhibition of α-glucosidase in vitro [7]. Experiments with sea buckthorn leaf preparations have also shown changes in lipid and glucose metabolism and reductions in inflammatory markers [8]. This composition makes the leaves relevant to cardiovascular research, but direct evidence remains limited. The biological argument primarily stems from studies of their principal flavonols and experiments with various sea buckthorn extracts. Isorhamnetin, quercetin, and kaempferol can influence oxidative and inflammatory pathways; some experiments have shown preservation of endothelial function or changes in eNOS and NO. Sea buckthorn preparations have also reduced oxidative injury, inflammation, and lipid abnormalities in cellular and animal models. These findings establish a reasonable line of investigation but do not demonstrate the cardiovascular effect of standardized leaf preparation in humans [5]. Clinical research has concentrated mainly on berries, juice, puree, and berry oil. Results for plasma lipids, indices of glycaemic control, inflammatory markers, and platelet activity have differed among studies, and most reported changes were small [9,10,11,12,13,14]. Another analysis also showed no overall benefit for blood pressure or glycaemic outcomes. Changes in circulating lipids were observed mainly among participants with abnormal lipid metabolism. The meta-analysis combined several sea buckthorn materials and formulations; however, its findings cannot be used to judge seed oil in isolation [15]. Evidence that can be applied directly to sea buckthorn leaves remains limited. Leaves differ from berries and oils in the proportions of flavonol glycosides, tannins, triterpenoids, carotenoids, and lipids. Greater total phenolic content alone does not establish stronger biological activity or clinical value. Żuchowski reviewed sea buckthorn phytochemistry and pharmacology across plant materials [4], and Y. Chen et al. reviewed cardiovascular evidence across sea buckthorn preparations [5]. The present review uses a documented search and selection process, separates findings for leaf preparations, isolated constituents, berries, and oils, and connects leaf chemistry with mechanisms, directness of evidence, and obstacles to clinical translation. The principal sea buckthorn materials, their characteristic phytochemical classes, and the reported cardiovascular and metabolic actions are summarized in Figure 1.

### Review Design, Search Strategy, and Study Selection

This article is a structured narrative review. Explicit eligibility criteria and a documented selection process were applied to increase the reproducibility and transparency of the review. The review was not designed as a systematic review, and no protocol was registered prospectively.

Two complementary PubMed searches covered records published from 1 January 2000 to 1 July 2026. The first combined names of sea buckthorn with terms describing plant materials, preparations, phytochemicals, cardiovascular and metabolic outcomes, vascular function, oxidative stress, inflammation, and safety. The second combines isorhamnetin, quercetin, and kaempferol with vascular and metabolic outcomes, redox balance, inflammation, platelet function, bioavailability, and microbial metabolism. Both searches were restricted to titles and abstracts, and the date limits were set before screening. Scopus was searched as a supplementary database and yielded no unique records. All publications included in the review were retrieved through PubMed. Reference lists were examined while the search queries were designed but did not contribute records to final selection. Author 1 screened all titles and abstracts and assessed the records retained for detailed review. Author 2 verified the screening decisions and resolved ambiguous cases. The authors did not perform two independent screenings, so no statistic for agreement between reviewers was calculated.

**Figure A1:**
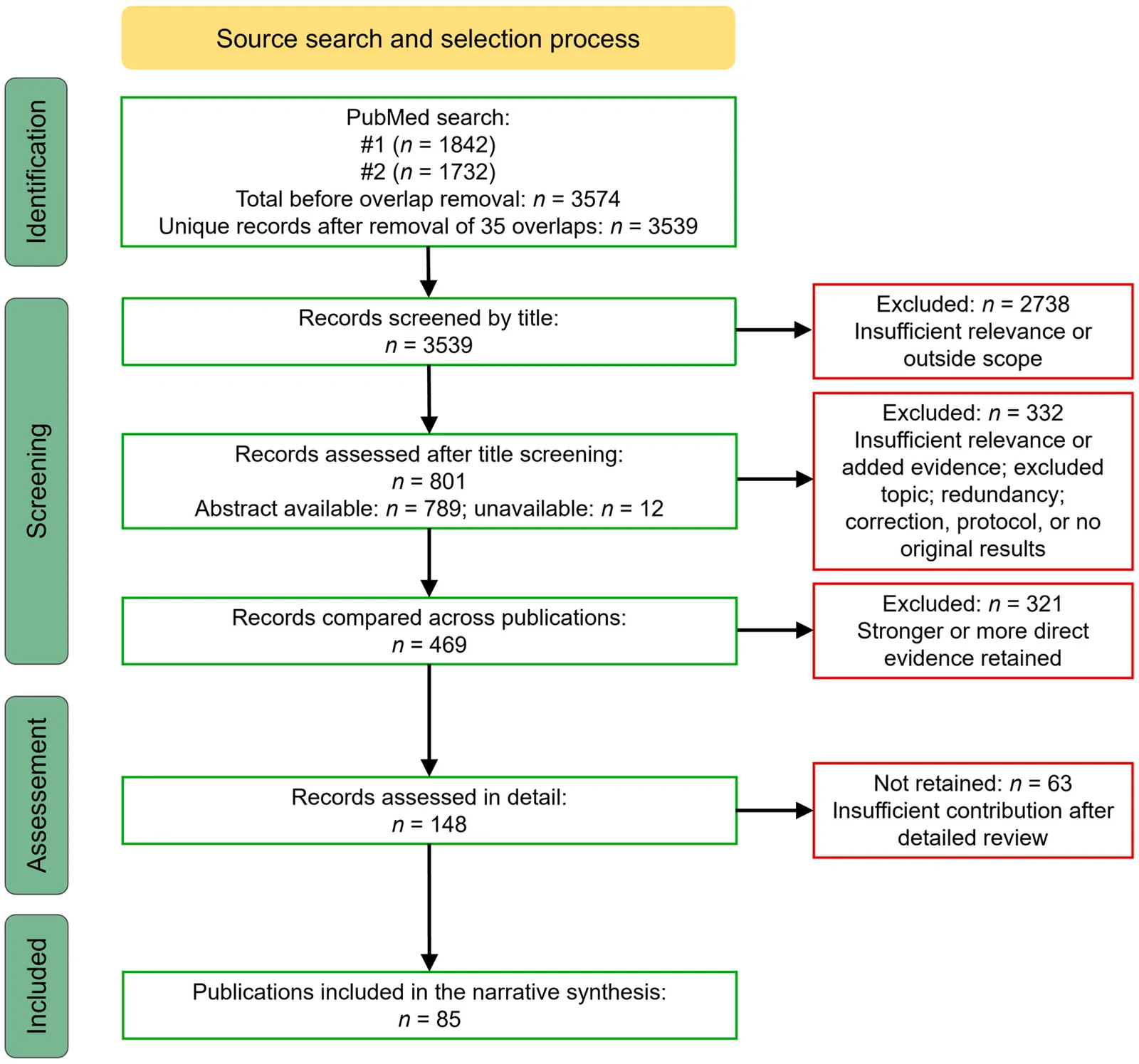
Flow diagram of the PubMed literature search and publication selection process. Scopus was used as a supplementary database and yielded no unique records.

The two PubMed searches returned 1842 and 1732 records. Removal of 35 overlapping records left 3539 unique records. Title screening excluded 2738 records and retained 801 for abstract assessment. Titles were excluded when they did not indicate sufficient relevance to cardiovascular or metabolic health, phytochemistry, pharmacokinetics, or safety; concerned disease areas outside the review scope; addressed agriculture, ecology, plant genetics, breeding, food technology, or animal feeding without a direct health question; or remained insufficiently relevant after manual assessment of a borderline title. Abstracts were available for 789 records. The 12 records without an abstract were assessed from the title and, when needed, the complete article. A sensitive abstract screen excluded 332 records because they lacked sufficient direct relevance or additional evidence, concerned an excluded topic, duplicated stronger evidence, or were corrections, protocols, or records without independent results. Global comparison of the 469 remaining records excluded 321 because a retained publication provided stronger, more direct, or less redundant evidence on the same issue. This left 148 records for detailed assessment. Human, in vivo, ex vivo, and in vitro studies and relevant reviews were eligible when they addressed the composition or standardization of sea buckthorn leaves, cardiovascular or metabolic outcomes, endothelial function, oxidative stress, inflammation, bioavailability, or safety. Detailed assessment retained 85 publications in English for the manuscript. A total of 63 records were not retained because they did not add sufficient evidence after examination of the complete article, repeated evidence already represented by a stronger source, or did not meet the language criteria. Figure A1 presents the selection process, and Table A1 provides the complete search queries and counts.

## 2. Differences in Phytochemical Composition Among Sea Buckthorn Materials

Each part of sea buckthorn has a distinct quantitative profile of bioactive constituents. Direct comparisons usually find more flavonoids and individually quantified phenolic compounds in leaves than in berries. Their concentrations can vary considerably with cultivar, harvest time, environmental conditions, processing and extraction methods, as well as with the way the results are reported [16,17,18]. A comparison of nine cultivars demonstrates the magnitude of this variation. The sum of the separately quantified phenolic compounds ranged from 1477.7 to 8709.0 mg/kg in leaves, whereas the corresponding values for berries were 76.1–205.2 mg/kg. Leaves also contained substantially more total flavonoids [6]. Considerable variation remains when leaves are examined individually. Studies of commercial teas and different germplasm resources have found substantial differences in total phenolics, total flavonoids, and individual flavonol profiles [16,17]. Flavonol glycosides and tannins are nevertheless commonly more abundant in leaves than in fruits [6,7,19]. The age of the harvested leaves may be particularly important for standardization. Nuclear magnetic resonance (NMR) fingerprints separated early collections, which contained relatively more polyphenols, from later collections, in which sugars and other carbohydrates made a greater contribution. Geographical location introduced additional, but smaller, differences [20]. Flavonol glycosides are characteristic constituents of sea buckthorn leaves. The group includes various 3-O-glycosides and 3,7-O-diglycosides of isorhamnetin, quercetin, and kaempferol [4]. Acylated structures occur as well. Tiliroside, a kaempferol glucoside acylated with *p*-coumaric acid, has been isolated directly from the leaves [21]. Zhao et al. later described a much wider range of kaempferol and isorhamnetin glycosides by liquid chromatography-mass spectrometry (LC–MS), including several compounds characterized for the first time. The study did not identify tiliroside as either exclusive to leaves or consistently dominant in the flavonoid profile of leaves [22]. Three main flavonol glycosides were reported in leaf infusions: isorhamnetin-3-O-glucoside-7-O-rhamnoside, isorhamnetin-3-O-rutinoside, and kaempferol-3-O-hexoside-7-O-rhamnoside. Stachyurin and casuarinin were the main ellagitannins; the processing at higher temperatures increased the levels of total phenolics and ellagitannins, but the resulting infusions showed lower antioxidant activity [23]. The phenolic acid profile also changes with the material and its preparation. Gallic, protocatechuic, and ellagic acids have been detected in raw leaves, extracts, and processed teas, but none occur at a uniform relative concentration across these materials [7,19]. Commercial leaf teas illustrate this variation. Ellagic acid had the highest concentration among the phenolic compounds quantified by He et al., yet its amount differed markedly between products [16]. A substantial share of the polyphenols in sea buckthorn leaves consists of tannins. Hydrolyzable ellagitannins coexist with condensed tannins, including catechins and procyanidins, although neither their amounts nor their proportions are uniform across plants [24,25]. Among 58 individual plants, the combined concentration of ten ellagitannins ranged from 42.5 to 109.1 mg/g of dry weight. Hippophaenin C, stachyurin, and casuarinin showed the highest mean concentrations, but each contributed differently to the tannin profile of individual plants [25]. In a separate study, fractionation of sea buckthorn leaves produced fractions enriched in casuarinin and its isomer casuarictin, after which casuarictin was isolated. The fractions and isolated casuarictin reduced tumor necrosis factor alpha (TNF-α) production in human whole blood stimulated with lipopolysaccharide (LPS) ex vivo and inhibited pancreatic amylase and lipase in vitro [26]. Woody tissues show a different balance of these compounds. Ethanol extracts from twigs contained more than ninefold the total catechin concentration measured in leaf extracts. The leaves still provided catechin, epicatechin, epigallocatechin, and type B procyanidin dimers, which contributed to the antioxidant activity of the extract [24]. Polyphenols are only one part of leaf chemistry. Pentacyclic triterpenes have been identified in the fruit, branches, and leaves, mainly within the oleanane and ursane families. Oleanolic and ursolic acids are among the compounds reported most often. Branch bark also yielded coumaroyl and caffeoyl esters of oleanolic and maslinic acids [27]. Liquid chromatography-high-resolution mass spectrometry (LC–HRMS) analysis of one leaf extract fraction led to the tentative identification of 19 triterpenoid glycosides. Their distribution differs between plant parts. In one direct comparison, fruit flesh contained approximately five times more triterpenoids than leaves, and ursolic acid represented 46% of the triterpenes measured [4]. Across the studies cited in this review, the leaf constituents most closely connected with the mechanisms discussed below are glycosides of isorhamnetin, quercetin, and kaempferol and the ellagitannins hippophaenin C, stachyurin, casuarinin, and casuarictin. These compounds are proposed markers for chemical standardization. No included study identified a constituent that caused the cardiovascular or metabolic effect of a complete leaf preparation.

Nine cultivars sampled at successive stages of ripening showed more vitamin E in leaves than in berries, although the amounts varied between cultivars and harvest dates [6]. Carotenoids followed the opposite pattern in six Romanian varieties. Leaves contained only 3.5–4.2 mg per 100 g of dry weight, in contrast to the markedly higher 53–97 mg per 100 g quantified in berries. Leaf carotenoids occurred exclusively in free forms and included lutein, violaxanthin, neoxanthin, zeaxanthin, β-carotene, and two cis β-carotene peaks [28]. Berries additionally provide vitamin C, tocopherols, and phytosterols, whose concentrations depend on cultivar, ripening stage, geographical origin, and post-harvest processing [18]. Comprehensive ultra-performance liquid chromatography with photodiode-array detection and quadrupole time-of-flight mass spectrometry (UPLC-PDA-Q/TOF-MS) profiling enabled the identification of 41 phenolic compounds in the analyzed berry samples. Isorhamnetin derivatives accounted for most of them, whereas quercetin and kaempferol glycosides were generally less abundant [29]. Glycosides of myricetin and syringetin have also been reported in some varieties [4].

Sea buckthorn seeds are used mainly as a source of oil. In seeds from two subspecies, total tocopherols and tocotrienols ranged from 84 to 318 mg/kg. α-, β-, γ-, and δ-tocopherol together accounted for 93–98% of this fraction. In whole berries, α-tocopherol alone accounted for 76–89% of the tocopherol and tocotrienol fraction [30].

Ethanol extracts from twigs contained more than nine times as much total catechins as leaf extracts and were particularly rich in protocatechuic acid and B-type procyanidins. Total flavonoid content was approximately twice as high in the leaf extract [24]. Work on four Romanian cultivars showed that the distinction between leaves and berries was functional as well as chemical. Extracts from leaves inhibited *Staphylococcus aureus* more strongly than extracts from berries of the same cultivar. Across the entire sample set, antioxidant activity closely followed total flavonoid yield (r = 0.96) [19]. A metabolomic comparison of leaves, berries, and commercial supplements examined whether individual chemical signals could distinguish these materials. Serotonin and tocopherol were associated more closely with leaf samples, together with signals assigned to luteolin-5-glucoside and isorhamnetin-3-rutinoside. These compounds remain proposed markers and require confirmation through targeted quantitative analysis [31]. Supplementary Table S1 summarizes reported quantitative and compositional findings for sea buckthorn leaves and products prepared from leaves in the cited studies. Values are presented with the material and reporting basis used in each source. NR indicates that the corresponding information was not reported. Results based on different materials, reporting bases, extracts, fractions, or assays were not pooled.

## 3. Mechanistic Evidence from Isolated Constituents

Unless the text states otherwise, the studies in this section concern isolated compounds or sources other than sea buckthorn leaves. They support biological plausibility and do not establish efficacy of a leaf preparation.

### 3.1. Isorhamnetin

Isorhamnetin is a principal flavonol in sea buckthorn leaves and is present mainly as glycosides, which shape the leaf’s flavonoid fingerprint. Among the reported conjugates are isorhamnetin-3-O-glucoside and isorhamnetin-3-O-rutinoside, commonly called narcissin, each imparting a subtly different polarity and stability to the aglycone. LC-MS profiling of Chinese material attributed eighteen of the fifty-seven detected flavonol glycosides to isorhamnetin [4,22]. During a six-hour incubation with angiotensin II, rat aortic rings lost much of their relaxation response to acetylcholine, while superoxide formation and p47phox expression rose. Isorhamnetin prevented each of these changes. Restricting NADPH oxidase activity maintained nitric oxide bioavailability within the vessel wall [32]. Isorhamnetin promoted Nrf2 nuclear translocation in HepG2 hepatocytes, driving transcriptional upregulation of heme oxygenase-1 and glutamate–cysteine ligase and increasing intracellular glutathione. Genetic or functional absence of Nrf2 prevented enzyme induction and eliminated protection from tert-butyl hydroperoxide-mediated oxidative injury [33]. Guo et al. examined isorhamnetin in rats with myocardial injury caused by isoproterenol and in H9c2 cells. In rats, treatment improved ejection fraction and fractional shortening and reduced fibrosis and circulating cardiac troponin I (cTnI) and CK-MB. In H9c2 cells, alpha-enolase (ENO1) expression and the concentrations of glucose and lactate declined, whereas PPARα, peroxisome proliferator-activated receptor gamma coactivator 1-alpha (PGC-1α), and ATP increased. These results suggest that the shift toward glycolysis was partly reduced, although fatty acid oxidation was not measured [34]. TNF-α increased apoptosis and upregulated ICAM-1, vascular cell adhesion molecule 1 (VCAM-1), E-selectin, NF-κB, and activator protein 1 (AP-1) in human umbilical vein endothelial cells (HUVECs). Isorhamnetin pretreatment attenuated these effects and increased eNOS expression [35]. Angiotensin II was also used in two mouse studies of cardiac remodeling. Isorhamnetin reduced ventricular hypertrophy and fibrosis and lowered the expression of transforming growth factor beta (TGF-β) and collagen type I α1 in the first study. In the second, it reduced susceptibility to atrial fibrillation, abnormal calcium handling, left atrial enlargement, and atrial fibrosis [36,37]. Available absorption data for isorhamnetin glycosides do not come from sea buckthorn. In a Caco-2 model, the aglycone was 2.6–4.6 times more permeable than glycosides isolated from Opuntia ficus-indica, and rat elimination half-lives were 0.64 h for the aglycone and 1.08 h for the Opuntia extract [38]. No human pharmacokinetic study has measured isorhamnetin exposure after administration of a chemically characterized sea buckthorn leaf preparation.

### 3.2. Quercetin

Quercetin (3,5,7,3′,4′-pentahydroxyflavone) appears in sea buckthorn leaves mainly in forms linked with various sugars, which shape the characteristic flavonol profile of the plant. Analysts typically find quercetin conjugated to: glucose, galactose or rutinose, which alter its chemical properties. LC–MS of leaves from Chinese plantations detected fifty-seven flavonol glycosides, sixteen of them based on quercetin [4,22]. Male spontaneously hypertensive rats received quercetin at 10 mg/kg daily for thirteen weeks. Over the course of treatment, the expected rises in blood pressure and heart rate were blunted, and the aorta recovered a clearer response to acetylcholine. Aortic eNOS was more abundant in untreated hypertensive rats than in normotensive controls, but its activity was lower. p47phox expression and superoxide production by NADPH oxidase were increased. Quercetin brought eNOS and p47phox expression closer to control levels, restored eNOS activity and lowered superoxide output [39]. Shen et al. showed that the vascular effects were dependent on AMP-activated protein kinase (AMPK): inhibiting this kinase abolished the protection against hypochlorous acid in mouse aortic rings treated with quercetin, 3′-O-methylquercetin, or quercetin-3-O-glucuronide. In human aortic endothelial cells, the same compounds increased phosphorylation of both AMPK and eNOS. The 5–10 μM concentrations used in vitro may exceed typical circulating exposure [40]. Quercetin and its 3-O-β-glucuronide countered part of the endothelial response to oxidized LDL and lysophosphatidylcholine. Both reduced adhesion molecule expression after either stimulus and prevented lysophosphatidylcholine from increasing caveolin-1, whereas oxidized LDL alone increased caveolin-1 mRNA [41]. In activated endothelial cells, the aglycone reduced ICAM-1 and VCAM-1 at both transcript and protein levels and lowered monocyte chemoattractant protein 1 (MCP-1) transcription. Its glucuronidated, sulfated and methylated metabolites were generally less potent. Surface VCAM-1 was an exception, as all three metabolites reduced its expression at 2 μmol/L [42]. Lipopolysaccharide produced a similar endothelial phenotype in human aortic cells, although the signaling pattern differed. Quercetin lowered E-selectin and ICAM-1 expression and curtailed oxidant production. Nrf2 activity increased, followed by higher expression of HO-1, NQO1 and glutamate–cysteine ligase. Because NF-κB activation remained intact, inhibition of that pathway cannot account for the reduced expression of adhesion molecules [43]. Endothelial monoculture did not fully predict the response to quercetin. Tumova et al. cultured human umbilical vein endothelial cells either alone or together with HepG2 hepatocytes or LHCN-M2 muscle cells. Quercetin increased HO-1 and lowered pyruvate dehydrogenase kinase 4 (PDK4) under each of these conditions, but the size of several responses changed when another cell type was present. HepG2 cells increased transendothelial glucose transport, and quercetin diminished the extent of that rise while leaving the integrity of the cell monolayer intact [44]. Thrombin impaired endothelial nucleotide handling by suppressing CD39/ATPase activity and promoted the release of ATP and ADP from activated platelets. In human umbilical vein endothelial cells, quercetin restored CD39/ATPase activity, and in thrombin-stimulated platelets, it reduced the release of ATP and ADP [45]. Its effects on platelets were not confined to the aglycone. Stainer et al. found that isorhamnetin and tamarixetin suppressed platelet aggregation with potency like that of quercetin. Both disrupted signaling downstream of glycoprotein VI and weakened the subsequent platelet response, which reduced thrombus formation under flow [46]. A study administering 730 mg of quercetin daily for 28 days reported a lowered blood pressure only in hypertensive patients, with the systolic pressure values dropping by 7 ± 2 mmHg, while both diastolic and mean arterial pressures decreased by 5 ± 2 mmHg. No comparable change was observed in participants with prehypertension. Plasma and urinary markers of oxidant stress also remained unchanged, so the reduction in blood pressure could not be linked to lower systemic oxidant stress [47]. In another trial, 88 patients who had experienced myocardial infarction had increased total antioxidant capacity after eight weeks of taking 500 mg of quercetin per day. Inflammatory markers and blood pressure, however, did not differ from those in the placebo group [48]. The umbrella review found only small, pooled decreases in systolic pressure and insulin. This pattern did not extend to diastolic pressure, while neither the lipid profile nor measures of glycaemic control and inflammation changed. Grading of Recommendations Assessment, Development and Evaluation (GRADE) placed confidence in these results between very low and, at best, moderate [49]. Quercetin’s beneficial effects must be understood in the context of its bioavailability. Nutritional quercetin is typically glycosylated, occurring as rutin or quercetin-4′-glucoside. Dietary quercetin glycosides can be hydrolyzed by beta-glucosidases in the epithelium of the small intestine; microbiota in the colon contributes to the metabolism of compounds that reach the large intestine [50,51]. It then undergoes extensive first-pass metabolism. Notably, all quercetin in plasma is present as conjugated metabolites, including glucuronides, sulfates, and methylated forms such as isorhamnetin [52]. Oral bioavailability in humans is variable and depends on the proper function of the intestinal epithelium and on metabolic and excretory processes [51]. In humans, the bioavailability of quercetin-3-glucoside did not differ significantly from that of quercetin-4′-glucoside [53]. Human studies of sea buckthorn flavonols also provide information on individual preparations. In a human meal study, no short-term changes were detected in markers of LDL oxidation, inflammation, homocysteine status, antioxidant capacity, or paraoxonase activity. Pharmacokinetic measurements nevertheless showed rapid uptake of sea buckthorn flavonols and suggested that co-ingested oil increased exposure at the higher dose [54]. After a sea buckthorn berry meal, circulating flavonol species were detected only in conjugated form, specifically as glucuronides derived from isorhamnetin and quercetin [55]. Findings from human interventions indicate that molecular form and delivery system are major determinants of quercetin exposure; characteristics of the accompanying meal, including its fat and fiber content, also modify absorption [56]. These findings are relevant to the development of rich in flavonols sea buckthorn formulations, including future standardized leaf products. Notably, the conjugate (quercetin-3-glucuronide) retains significant bioactivity, although often slightly lower than that of the free aglycone. Some conjugates can slowly deconjugate in tissues or at sites of inflammation. This process is facilitated by β-glucuronidase enzymes released by immune cells. As a result, free quercetin may be locally regenerated. Its methylated metabolite, isorhamnetin, may also contribute to these effects. In isolated vessels exposed to endothelin-1, 3′-O-methylquercetin prevented eNOS uncoupling [40]. Although the experimental data supports vascular and antioxidant effects of quercetin, their direct relevance to sea buckthorn leaves remains uncertain. No human pharmacokinetic study has established exposure to quercetin glycosides or their metabolites after administration of a chemically characterized sea buckthorn leaf preparation.

### 3.3. Kaempferol

Kaempferol (3,5,7,4′-tetrahydroxyflavone) is another prominent flavonol in sea buckthorn leaves. Kaempferol is present in sea buckthorn leaves in several glycosylated forms. Zhao et al. identified 14 kaempferol glycosides by LC–MS, alongside 18 glycosides of isorhamnetin and 16 of quercetin. Overall, 57 flavonoids were identified. Nicotiflorin (kaempferol-3-O-rutinoside) and astragalin (kaempferol-3-O-glucoside) were also identified. Available data do not justify describing nicotiflorin as a universally predominant phenolic in sea buckthorn leaves [22]. Tiliroside, a *p*-coumaroylated kaempferol glucoside, has been isolated from sea buckthorn leaves [21]. More recent analyses reveal many kaempferol glycosides in the leaves, with tiliroside not standing out as a dominant marker [22]. Kaempferol shows cardioprotective actions like quercetin. A central vascular mechanism involves the regulation of the Nrf2/HO-1 and NF-κB pathways. Across HUVEC and mouse vascular injury experiments, kaempferol produced a shift toward antioxidant and anti-inflammatory signaling. Treatment enhanced Nrf2/HO-1 defenses while reducing NF-κB activation and the cytokines TNF-α and IL-6, with corresponding attenuation of endothelial and vascular injury [57]. The antioxidant and anti-inflammatory effects of kaempferol, together with its protective effects on the endothelium are relevant to the phytochemical profile of sea buckthorn leaves rich in kaempferol. Kaempferol also acts directly on the vascular system. In isolated artery studies, it induces relaxation independent of the endothelium by acting on vascular smooth muscle. Calcium channel modulation or potentiation of nitric oxide signaling possibly modulates these effects [58]. Moreover, a recent study in an atherosclerosis model found that kaempferol promoted plaque stabilization and vascular repair. It increased the thickness of the fibrous cap in plaques and facilitated eNOS recovery in the endothelium adjacent to plaque [59]. This suggests kaempferol prevents endothelial damage and can help restore endothelial function after injury. In rats receiving Nω-nitro-L-arginine methyl ester (L-NAME) for five weeks, concomitant oral administration of kaempferol at either 20 or 40 mg/kg partially attenuated the development of hypertension and alleviated left-ventricular dysfunction and hypertrophy. Kaempferol also improved vascular reactivity and aortic wall morphology and reduced abnormalities in redox and inflammatory markers. These effects were accompanied by suppression of changes induced by L-NAME in the expression or phosphorylation of proteins associated with TNF-α signaling in cardiac and vascular tissues [60]. Kaempferol lowered reactive oxygen species (ROS) production in macrophages exposed to LPS. NLRP3 (NOD-, LRR- and pyrin domain-containing protein 3) activation and pyroptosis decreased, while glutathione and HO-1 levels increased; inhibition of Nrf2 attenuated these responses [61]. Taken together, these findings indicate substantial biological activity of kaempferol and its preparations. However, most published studies derive from isolated compounds, animal models, or non-standardized preparations; and it remains unknown whether comparable exposure and effects can be achieved with sea buckthorn leaves in humans.

### 3.4. Ellagitannins and Urolithins

Sea buckthorn leaves contain ellagitannins that include hippophaenin C, stachyurin, casuarinin, and casuarictin [25,26]. Hydrolysis can release ellagic acid, which specialized bacteria in the human gut can convert to urolithins [62]. This microbial pathway has not been demonstrated after consumption of sea buckthorn leaves. Urolithin A reduced lesion burden and endothelial inflammatory activation in experimental atherosclerosis [63]. This result does not show that leaf preparation produces sufficient urolithin exposure in humans. A human pharmacokinetic study of a chemically characterized leaf preparation is needed before this pathway can support a claim about clinical translation.

Extracts rich in ellagitannins have also been reported to reduce NF-κB activity and vascular inflammation. Ellagitannins can bind to enzymes such as ACE and lipoxygenase, thereby reducing their activity. This may influence cardiovascular function, although direct clinical data is lacking [64]. Condensed tannins are present in sea buckthorn leaves and twigs. They occur mainly as B-type procyanidins built from catechin and epicatechin. Twig extract scavenged 2,2-diphenyl-1-picrylhydrazyl (DPPH) radicals over the range of 0.5–50 µg/mL. At 50 µg/mL, the reduction was about 70% [24]. In a separate study, sea buckthorn leaves were fractionated to enrich casuarinin and its isomer casuarictin, after which casuarictin was isolated. The resulting fractions and isolated casuarictin decreased TNF-α production ex vivo in human whole blood stimulated with LPS and inhibited pancreatic amylase and lipase in vitro [26]. These results show that ellagitannin-rich leaf fractions can affect inflammatory responses and digestive enzymes. Gallic acid and related compounds have also been found in leaf preparations. Their amounts differ with the plant material and the method of processing [7,16,19]. Sea buckthorn leaf extract was fractionated and tested against α-glucosidase. At 5 µg/mL, the butanol fraction produced the strongest inhibition. Casuarinin was also active, with an IC50 of 21 µM [4].

### 3.5. Leaf Fractions and Other Constituents

**Figure 2:**
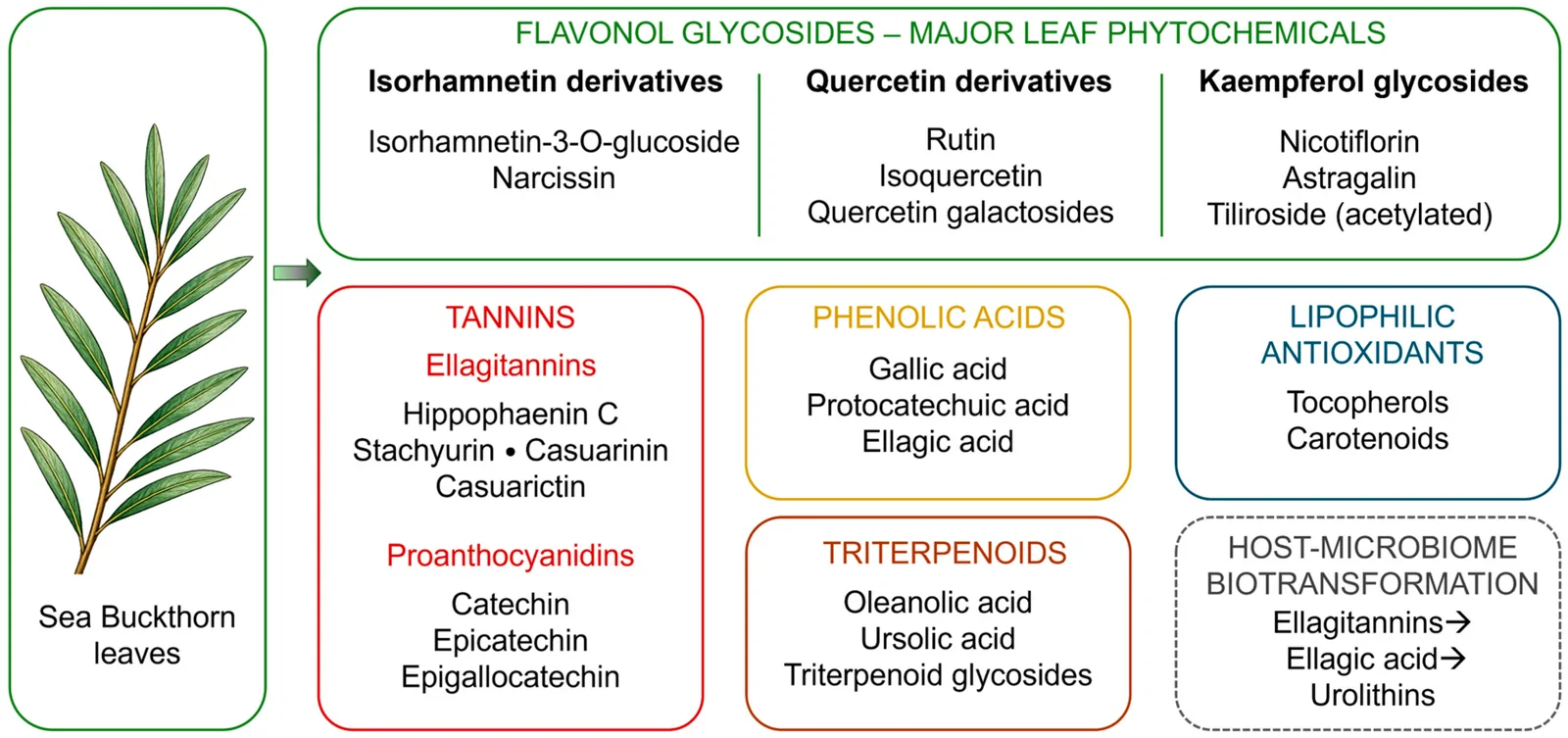
Major phytochemical classes reported in sea buckthorn leaves. Representative flavonol glycosides, ellagitannins, proanthocyanidins, phenolic acids, lipophilic antioxidants, and triterpenoids are shown. Ellagitannins may undergo hydrolysis to ellagic acid followed by conversion to urolithins by the gut microbiota. This pathway is supported by general microbiome studies and has not been demonstrated after consumption of sea buckthorn leaves in humans.

Acylated flavonol glycosides that contain menthiafolic acid have been reported in sea buckthorn leaves. A study identified two such compounds as derivatives of kaempferol and isorhamnetin, without examining their biological activity. Sea buckthorn leaves contain pentacyclic triterpenoids and their glycosides: 19 triterpenoid glycosides were tentatively identified in a leaf fraction, although their individual activity was not tested [4]. Coumaroyl and caffeoyl esters of oleanolic and maslinic acids were isolated from branch bark. Their effects on NO production in activated macrophages and DPPH radical scavenging were assessed in vitro [27]. The main phytochemical classes reported in sea buckthorn leaves, with emphasis on flavonol glycosides, are summarized in Figure 2. Their occurrence in leaves, proposed cardiovascular and metabolic mechanisms, and translational relevance are compared in Table 1.

## 4. Preclinical Evidence by Tested Material

### 4.1. Leaf Extracts and Fractions Prepared from Leaves

Sea buckthorn leaves have been tested in metabolic, vascular, and ex vivo models. In mice fed a diet rich in fat, a complete leaf extract and a fraction rich in flavonol glycosides limited adiposity, hepatic steatosis, insulin resistance, and disturbances in lipid metabolism [8]. Fermented leaf tea reduced weight gain and hepatic lipid accumulation in rats with hyperlipidemia; hepatic LKB1 and AMPK activity increased, and ACC1 and SREBP1c expression decreased [66]. In vitro, leaf extract inhibited the formation of advanced glycation end products and collagen crosslinking more strongly than berry extract and showed greater ABTS and DPPH scavenging activity [67]. Leaf extracts also showed antioxidant activity in cell assays [24]. In rats exposed to acute hypobaric hypoxia, leaf extract reduced pulmonary edema, leakage across the vascular barrier, cytokines, VEGF, and catecholamines and preserved relaxation of pulmonary arteries in response to acetylcholine [68]. In human whole blood ex vivo, extracts from leaves and twigs reduced platelet responses to ADP and collagen, with the clearest effect at 50 µg/mL [69]. These studies show direct biological activity of preparations from leaves, but the preparations differ in composition and no study replicated the effects of one chemically defined preparation.

### 4.2. Isolated Constituents and Preparations with Incomplete Characterization

Flavonoid compounds obtained from sea buckthorn protected endothelial cells exposed to H_2_O_2_ through a pathway that involved PI3K, AKT, and eNOS [70]. The plant part and quantitative composition were not described well enough to attribute this result to leaves. Isolated isorhamnetin reduced injury caused by oxidized LDL in endothelial cells [65] and reduced ischemia and reperfusion injury in isolated rat hearts [71]. In Zucker diabetic fatty rats, administration of a sea buckthorn preparation reduced hyperglycemia and sorbitol accumulation, but the tested plant material was not identified clearly enough to treat the study as evidence from leaves [72]. A pressed sea buckthorn preparation improved blood pressure and selected metabolic measures in spontaneously hypertensive rats, but the plant part and quantitative composition were not reported sufficiently [5]. Total flavones reduced thrombogenesis in mice, but the botanical source of the flavone preparation was not established [73]. These studies cannot be presented as direct evidence for a sea buckthorn leaf preparation.

### 4.3. Berries, Oils, and Other Plant Materials

Berry polyphenols reduced aortic ICAM-1 and LOX-1 expression, circulating lipids, inflammatory cytokines, and oxidative stress measures in rats with hyperlipidemia [74]. Sea buckthorn pulp oil reduced myocardial injury after ischemia and reperfusion in rats and altered Akt, eNOS, IKKβ, NF-κB, apoptosis, and oxidative injury measures [75]. These findings provide context for sea buckthorn research and cannot establish the effects of leaves. No study compared one chemically defined leaf preparation with berries, an established drug, and an established botanical comparator in the same validated animal model using a hard cardiovascular or metabolic outcome.

## 5. Human Evidence by Sea Buckthorn Preparation

No controlled human trial has evaluated a chemically characterized sea buckthorn leaf preparation for cardiovascular or metabolic outcomes. The studies of berries, purée, juice, and oils below provide clinical context and cannot establish efficacy of leaves.

### 5.1. Berries, Purée, and Juice

In healthy adults, sea buckthorn berries have usually had little effect on the lipid profile. The trial enrolled 229 participants and found no difference from placebo in total, LDL, HDL, or triglyceride levels. Plasma flavonol concentrations increased [9]. A different response was seen in overweight women with a poorer metabolic profile at baseline. In this subgroup, 30 days of dried berry consumption reduced triglyceride and VLDL concentrations. The isolated berry phenolic extract produced no comparable change [10]. Across 15 randomized trials, changes in the lipid profile were modest and clearer in participants with abnormal lipid concentrations [15]. However, in 111 participants with hypercholesterolemia, 90 days of sea buckthorn puree did not change total cholesterol, LDL cholesterol, or triglycerides. Diastolic pressure was slightly lower, but the difference from placebo was not significant [14]. The lipid response, therefore, appears to depend on the preparation and the metabolic status of the participants. Whole berries and juices have generally had little effect on blood pressure in healthy or normotensive participants [5]. A meta-analysis of randomized trials also found no significant overall effect [15]. Evidence in patients with hypertension remains limited, and no benefit has been established for a particular berry preparation. Changes in glucose regulation have also been small. In a crossover trial of 38 participants with impaired glucose regulation, five weeks of 90 mL of fruit puree per day reduced median fasting glucose by 0.14 mmol/L. During placebo, it increased by 0.07 mmol/L. Glycated serum protein and the two-hour postprandial glucose area under the curve (AUC) remained unchanged [12]. Short studies in healthy participants have found no meaningful change in fasting glucose or insulin, and the meta-analysis showed no overall effect on blood glucose [15]. Acute responses may differ. In overweight or obese men, a sea buckthorn meal did not lower postprandial glucose, but the rise in insulin was smaller and occurred later than after the control meal [76]. Evidence concerning inflammation is limited. In the trial of 229 healthy adults, high-sensitivity C-reactive protein (CRP) decreased from baseline in the sea buckthorn group. This change was not associated with the increase in plasma flavonols [9]. Most clinical studies of berry preparations did not measure individual cytokines. The available CRP finding, therefore, does not establish a consistent anti-inflammatory effect in humans. Clinical data for vascular effects also remains limited. In healthy men, sea buckthorn juice increased plasma antioxidant capacity and delayed LDL oxidation [11]. Berries added to a meal rich in fat also reduced the postprandial rise in triglycerides [77].

### 5.2. Oils and Lipid Extracts

Pulp oil and seed oil have different fatty-acid profiles. The proportions of palmitoleic, oleic, linoleic, and α-linolenic acids vary between them [78]. In the crossover trial of overweight women, seed oil showed a slight tendency to reduce total and LDL cholesterol [10]. A separate crossover study included 12 healthy men. Five grams of berry oil per day for four weeks reduced platelet aggregation after ADP stimulation ex vivo. Plasma lipids and glucose remained unchanged [13]. In another study, 86 patients with chronic coronary syndrome received a seed lipid extract in addition to standard treatment. After three months, systolic pressure, LDL cholesterol, and CRP were slightly lower [79]. The modest or inconsistent findings may reflect differences in preparation, incomplete chemical characterization, short interventions, small samples, enrollment of healthy or normolipidemic participants, results confined to subgroups, and reliance on surrogate biomarkers. Leaf preparations differ chemically from berries and oils, but greater phenolic content alone does not predict greater systemic exposure or clinical efficacy. The existing studies of berries and oils neither predict benefit nor demonstrate lack of benefit for leaves.

### 5.3. Direct Evidence from Leaves and Clinical Interpretation

**Figure 3:**
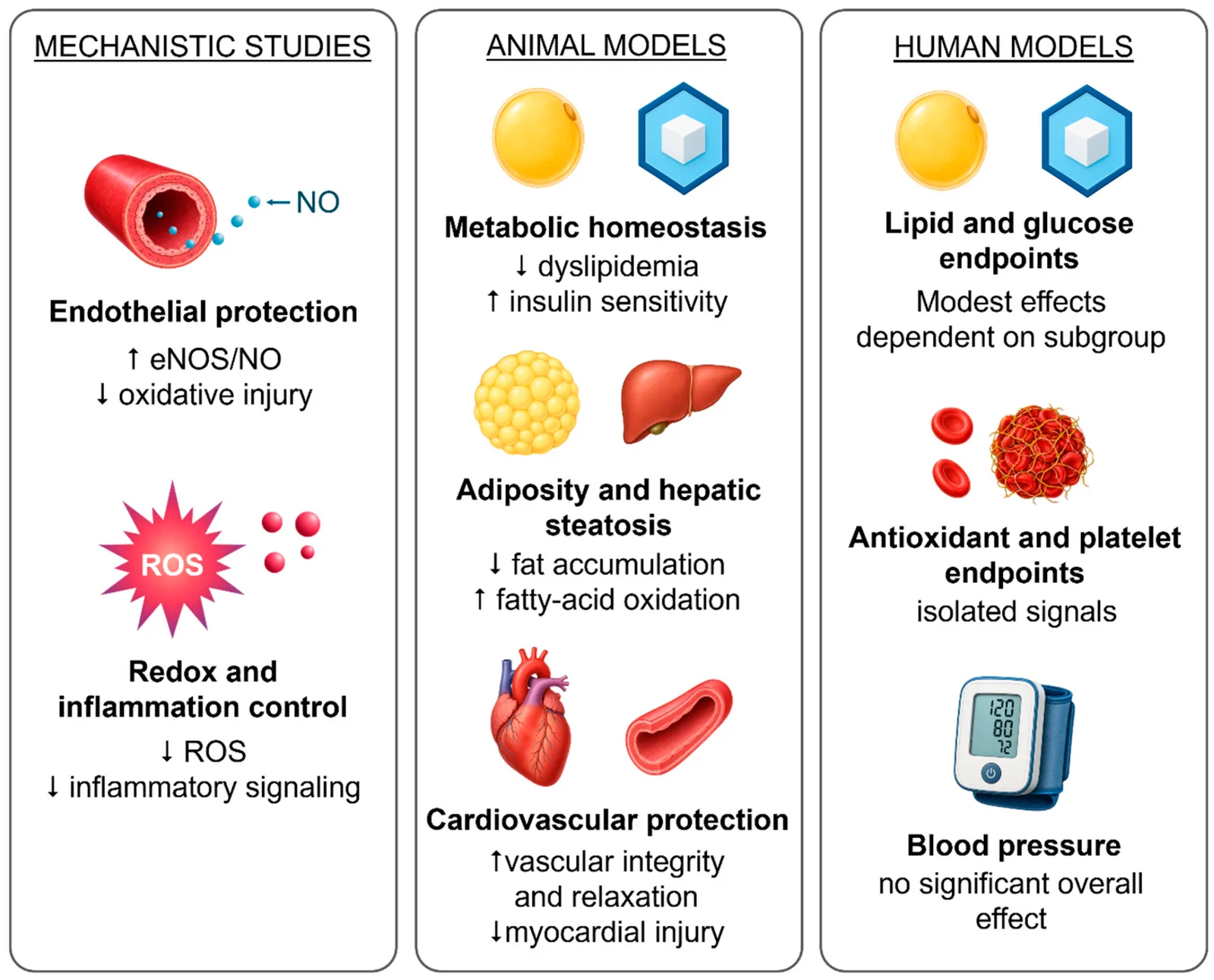
Translational overview of cardiovascular and metabolic evidence for sea buckthorn. Mechanistic studies support preservation of eNOS/NO signaling and attenuation of oxidative and inflammatory pathways. Animal models show improvements in metabolic homeostasis, adiposity, hepatic steatosis, and vascular or myocardial injury. Human findings are less consistent and arise mainly from berries, puree, juice, or oil preparations. Lipid and glucose responses are modest and vary by subgroup, antioxidant and antiplatelet findings are isolated, and pooled randomized evidence shows no significant overall effect on blood pressure. No cardiovascular randomized trial has evaluated standardized leaf preparations.

No human pharmacokinetic study has quantified flavonol or ellagitannin metabolites after administration of a sea buckthorn leaf preparation. No study in humans has assessed responses across several doses or controlled safety of a chemically characterized leaf preparation. The animal and ex vivo studies justify additional research on lipid metabolism, glucose regulation, vascular function, and hemostasis. They do not establish human effects and do not justify an efficacy trial before replication of the same preparation in more than one relevant animal study. Figure 3 summarizes this gap, and Table 2 compares the available human studies by preparation.

## 6. Safety and Tolerability

Ethanol extracts prepared from leaves and twigs caused little toxicity in assays performed in cultured cells. No acute cytotoxic or hemolytic effects were observed at concentrations of up to 50 µg/mL, and normal human fibroblasts were only minimally affected [24]. The findings in animals were similarly favorable. For an aqueous leaf extract, the oral LD50 in rats exceeded 10 g/kg. Administration of 100 mg/kg each day for 30 days produced no important changes in hematological or biochemical measurements. Alterations in liver or kidney weight were reported only in separate 14-day experiments in which much higher doses of 1 or 2 g/kg per day were used [80]. Leaf extracts obtained through conventional extraction and extraction under pressure also showed little cytotoxicity in the in vitro models examined by Pap et al. [81]. In vitro, the direction of the leaf extract’s effect on DNA depended on concentration: lower exposure was protective, whereas higher exposure became damaging. The authors linked this reversal to trace-metal-mediated hydroxyl radical formation, making extract concentration and metal content relevant formulation variables; these findings do not demonstrate genotoxicity in vivo [82]. In published human studies evaluating berries, juice, puree, and berry oil, short-term tolerability has generally been favorable, with no serious treatment-related safety signals reported [9,11,12,13,14]. These findings apply only to the preparation tested and should not be extrapolated to standardized extracts from leaves. Animal toxicology data are available for sea buckthorn oil [83], whereas clinical safety data for chemically standardized leaf preparations, defined polyphenol doses, long-term administration, pregnancy, and drug interactions remain limited. Although the available experiments with leaf extracts have raised a few toxicological concerns, evidence from cultured cells cannot establish safety in humans [81]. Carotenodermia is a possible, but harmless, consequence of consuming unusually large amounts of foods rich in carotenoids. It appears as a yellow or orange discoloration of the skin. Since sea buckthorn berries and their oil contain substantial amounts of β-carotene, prolonged and excessive intake could conceivably produce this effect [84]. It has not been described in the short clinical trials of sea buckthorn. Should it occur during sustained heavy consumption, the discoloration would be expected to fade after the intake is reduced. The unsaturated fatty acids present in sea buckthorn oils may influence several processes involved in cardiovascular and metabolic regulation [78]. Whether these effects are strong enough to alter the action of medicines in humans remains uncertain. Available clinical studies have not identified interactions between sea buckthorn preparations and antihypertensive medicines or medicines used to lower blood glucose [12,15]. Berry oil reduced platelet aggregation in one small human study, and extracts from leaves and twigs altered platelet reactivity in human whole blood ex vivo [13,69]. Flavonoids isolated from berries also inhibited platelet activity [85]. These observations do not establish clinical interaction. Future studies of leaf preparations should record the use of antiplatelet and anticoagulant medicines, define bleeding outcomes before recruitment, and monitor relevant measures of hemostasis. Table 3 presents the authors’ qualitative narrative appraisal of directness, consistency, replication, and chemical characterization. The labels were not generated with GRADE or another validated instrument and should not be interpreted as formal ratings of certainty.

## 7. Conclusions

Sea buckthorn leaves contain a diverse mixture of phytochemicals, but current evidence does not establish a cardiovascular or metabolic benefit in humans or identify a constituent responsible for the effects of complete leaf preparation. Studies of isolated isorhamnetin, quercetin, kaempferol, and urolithin A support biological plausibility; studies of berries and oils provide clinical context only. No controlled human trial or human pharmacokinetic study of leaf preparation is available. Research should begin with one leaf batch characterized for isorhamnetin-3-O-glucoside-7-O-rhamnoside, isorhamnetin-3-O-rutinoside, representative quercetin and kaempferol glycosides, hippophaenin C, stachyurin, and casuarinin. The same preparation should then be compared with berries, an established drug, and an established botanical comparator in a validated animal model with a hard cardiovascular or metabolic outcome. Fractions prepared from the same batch should test whether the response follows common flavonols, ellagitannins, or a less common constituent. A positive result should be reproduced in more than one relevant animal study before research in humans begins. Human research should proceed through pharmacokinetics, assessment of several doses, safety over a defined period with hemostatic monitoring, and an efficacy trial only if exposure and safety findings justify it. Current evidence does not show a clinical or economic advantage over common dietary sources of similar flavonoids.

## Figures and Tables

**Table 1:** Translational matrix for major phytochemical groups reported in sea buckthorn leaves.

Leaf Constituent	Occurrence in Leaves	Experimental Mechanisms	Translational Interpretation
Isorhamnetin derivatives [4,22,32,33,35,65]	One of the main leaf flavonols; present mainly as glycosides.	Evidence from studies of isolated isorhamnetin: preservation of eNOS and NO signaling; lower NADPH oxidase activity and ROS; Nrf2/HO-1 activation; attenuation of inflammatory and apoptotic pathways.	Mechanistic and preclinical support is substantial, but human exposure, effective dose, and clinical efficacy remain unknown.
Quercetin derivatives [22,32,39,40,43,47]	Common leaf glycosides include isoquercetin, rutin, and galactosides.	Evidence from studies of isolated quercetin and its metabolites: higher eNOS activity and NO availability; Nrf2/HO-1 activation; lower oxidant production, adhesion molecules, and inflammatory signaling.	Human evidence for quercetin cannot be attributed to sea buckthorn leaves without formulation and bioavailability studies.
Kaempferol glycosides [4,21,22,57,58,60]	Multiple glycosides occur in leaves, including astragalin, nicotiflorin, and tiliroside.	Evidence from studies of isolated kaempferol: Nrf2/HO-1 activation; attenuation of NF-kB/TNF-α signaling; vascular relaxation; improvement of vascular reactivity and cardiac injury in animals.	The achievable exposure to kaempferol metabolites from leaf preparations and their contribution to clinical effects are unresolved.
Ellagitannins [25,26,62,63]	Hippophaenin C, stachyurin, casuarinin, and casuarictin are prominent leaf tannins.	Evidence combines analyses of leaf composition with general studies of ellagic acid metabolism and experimental studies of urolithins; the complete pathway has not been demonstrated after administration of a sea buckthorn leaf preparation.	Translation depends on processing, bioavailability, and individual microbiota metabotype; a pharmacokinetic study of leaf preparations is required.

**Table 2:** Human evidence by preparation: cardiovascular and metabolic endpoints.

Population/Evidence Base	Preparation, Dose, Duration	Main Findings	Key Limitation
Healthy adults (*n* = 229) [9]	Frozen berries, 28 g/day 3 months	Total, LDL, HDL cholesterol and triglycerides were unchanged. Plasma quercetin and isorhamnetin increased.	Healthy, mainly normolipidaemic cohort; no direct vascular function endpoint.
Overweight women (*n* = 80) [10]	Dried berries; berry phenolic extract; seed oil; bilberries 30 days per intervention	In the higher-risk subgroup, berries reduced triglycerides and VLDL; seed oil showed a downward trend in total and LDL cholesterol. The phenolic extract did not improve lipids.	Trends dependent on subgroups; many individual changes were not significant after correction for multiple comparisons.
Impaired glucose regulation (*n* = 38) [12]	Fruit puree, 90 mL/day 5 weeks per intervention	Median fasting glucose changed by −0.14 mmol/L with puree and +0.07 mmol/L with placebo. Glycated serum protein and 2-h AUC did not differ.	Small effect on one glycaemic endpoint; short intervention.
Direct evidence from leaves	No controlled human trial of a chemically characterized leaf tea or leaf extract	No estimate of cardiovascular or metabolic efficacy is available.	No human pharmacokinetic study, assessment of several doses, or controlled safety study of a leaf preparation.
Healthy men (*n* = 20) [11]	Sea buckthorn juice drink, 300 mL/day 8 weeks	LDL oxidation susceptibility decreased moderately. No significant changes in plasma lipids, platelet aggregation, or ICAM-1 were reported.	Small sample; no FMD, PWV, or clinical cardiovascular endpoint.
Healthy normolipidaemic men (*n* = 12) [13]	Berry oil, 5 g/day 4 weeks per intervention	ADP-stimulated platelet aggregation decreased. Plasma lipids and glucose were unchanged.	Preliminary crossover study; very small sample; ex vivo endpoint not linked to clinical outcomes.
Adults with hypercholesterolaemia (*n* = 111) [14]	Fruit puree, 90 mL/day 90 days	Total cholesterol, LDL cholesterol, and triglycerides were unchanged. The reduction in diastolic pressure was not significant; hsCRP decreased moderately.	Largely null primary lipid findings; no significant blood-pressure effect.
Meta-analysis (15 RCTs) [15]	Mixed sea buckthorn preparations; varied doses and intervention durations	Pooled lipid outcomes improved modestly, mainly in participants with abnormal lipid metabolism. There was no significant overall effect on blood pressure, glucose, or BMI.	High heterogeneity; mixed preparations and populations.
Chronic coronary syndrome (*n* = 86) [79]	Seed lipid extract, 1000 mg twice daily, added to standard therapy 3 months	From baseline, systolic pressure decreased by 2.9 mmHg, LDL cholesterol by 0.3 mmol/L, and CRP by 1.0 mg/L.	Uncontrolled pilot study; background medication and regression to the mean cannot be separated from the intervention; industry involvement.

ADP, adenosine diphosphate; AUC, area under the curve; BMI, body mass index; CRP, C-reactive protein; FMD, flow-mediated dilation; HDL, high-density lipoprotein; hsCRP, high-sensitivity C-reactive protein; ICAM-1, intercellular adhesion molecule 1; LDL, low-density lipoprotein; n, number of participants; PWV, pulse wave velocity; RCT, randomized controlled trial; VLDL, very-low-density lipoprotein.

**Table 3:** Sea buckthorn (*Hippophae rhamnoides*) leaves: phytochemical profile and cardiovascular evidence.

Domain	Summary	Limitations	Authors’ Narrative Assessment
Dominant phytochemicals [4,7,16,19,24,25,28]	Leaves generally contain more total polyphenols and flavonoids than berries. Major groups include glycosides of isorhamnetin, quercetin, and kaempferol; ellagitannins and proanthocyanidins; pentacyclic triterpenoids; tocopherols; and smaller amounts of carotenoids.	Concentrations vary with cultivar, harvest time, processing, extraction, and reporting method. Marker compounds are not consistently standardized.	High
Mechanistic studies [4,5,32,35,43,51,62,65,70,71]	The evidence converges on preservation of NO and eNOS signaling, lower oxidative stress through Nrf2/HO-1 and inhibition of pro-oxidant pathways and reduced inflammatory signaling through pathways related to NF-kB/MAPK. Conversion of ellagitannin metabolites by the gut microbiota is plausible.	Many data come from isolated flavonols or non-leaf preparations. Experimental concentrations and aglycones may not reflect circulating human metabolites.	Moderate–High
Animal studies [8,66,68]	Leaf extracts improved adiposity, insulin resistance, hepatic steatosis, lipid metabolism, inflammation, and oxidative injury in several models. Vascular and organ protection has also been reported in stress paradigms.	Metabolic endpoints are more common than direct measures of arterial function, stiffness, or atherosclerotic burden. Extract composition is often incompletely reported.	Moderate
Endothelial and vascular cell evidence [32,35,43,65,70]	Studies of isolated isorhamnetin and quercetin, and studies of incompletely characterized sea buckthorn flavonoid preparations reported preservation of NO signaling, lower ROS, and attenuation of adhesion and inflammatory pathways. These findings do not demonstrate an effect of a leaf preparation.	Cell concentrations may exceed achievable plasma exposure. Metabolites and glycosides can differ in activity from the aglycones used experimentally.	Moderate–High
Human evidence	No controlled human trial has evaluated a chemically characterized sea buckthorn leaf preparation for cardiovascular or metabolic outcomes.	Clinical cardiovascular evidence comes mainly from berries, puree, juice, and oils. Efficacy and dose-response data for leaf preparations are absent.	Low
Safety [24,80]	Leaf and twig extracts showed low toxicity in selected cell assays. In rats, the oral LD50 of an aqueous leaf extract exceeded 10 g/kg, and 100 mg/kg/day for 30 days was well tolerated.	Clinical safety data are unavailable for chemically standardized leaf preparations, defined polyphenol doses, long-term use, pregnancy, or drug interactions.	Moderate
Standardization considerations [4,7,16,19,24,25,28]	Leaf preparations should be described as mixtures of flavonol glycosides and tannins. Candidate markers include isorhamnetin-3-O-glucoside or rutinoside, quercetin glycosides, and major ellagitannins such as hippophaenin C, stachyurin, and casuarinin.	Extraction, reporting as aglycone equivalents, and marker profiling require harmonization. Products labelled as total flavones require a documented botanical source and chemical composition.	Moderate

Authors’ narrative assessment: qualitative appraisal based on study design, directness to leaf preparations, consistency, replication, and chemical standardization. The labels are not GRADE ratings or results from another validated instrument. eNOS, endothelial nitric oxide synthase; HO-1, heme oxygenase 1; LD50, median lethal dose; MAPK, mitogen-activated protein kinase; NF-κB, nuclear factor kappa B; NO, nitric oxide; Nrf2, nuclear factor erythroid 2-related factor 2; ROS, reactive oxygen species.

## Data Availability

No new data were created or analyzed in this study. Data sharing is not applicable to this article.

## Supplementary Materials

The following supporting information can be downloaded at: https://www.mdpi.com/article/10.3390/nu18183009/s1. Table S1: Reported composition of sea buckthorn leaves and leaf preparations [6,7,8,16,17,19,20,21,22,23,24,25,26,28,31,69,81].

## Abbreviations

The following abbreviations are used in this manuscript:

ABTS	2,2′-azino-bis(3-ethylbenzothiazoline-6-sulfonic acid)
ACC1	acetyl-coenzyme A carboxylase 1
ADP	adenosine diphosphate
AKT	protein kinase B
AMPK	AMP-activated protein kinase
ApoE	apolipoprotein E
CK-MB	creatine kinase myocardial band
CRP	C-reactive protein
cTnI	cardiac troponin I
DPPH	2,2-diphenyl-1-picrylhydrazyl
ENO1	alpha-enolase
eNOS	endothelial nitric oxide synthase
HDL	high-density lipoprotein
HO-1	heme oxygenase 1
hsCRP	high-sensitivity C-reactive protein
ICAM-1	intercellular adhesion molecule 1
IKKβ	IκB kinase beta
LC–MS	liquid chromatography–mass spectrometry
LDH	lactate dehydrogenase
LDL	low-density lipoprotein
LKB1	liver kinase B1
LPS	lipopolysaccharide
NADPH	reduced nicotinamide adenine dinucleotide phosphate
NLRP3	NOD-, LRR-, and pyrin domain-containing protein 3
NF-κB	nuclear factor kappa B
NO	nitric oxide
Nrf2	nuclear factor erythroid 2-related factor 2
NQO1	NAD(P)H:quinone oxidoreductase 1
p38 MAPK	p38 mitogen-activated protein kinase
PGC-1α	peroxisome proliferator-activated receptor gamma coactivator 1-alpha
PI3K	phosphoinositide 3-kinase
ROS	reactive oxygen species
SREBP1c	sterol regulatory element-binding protein 1c
TGF-β	transforming growth factor beta
TNF-α	tumor necrosis factor alpha
VCAM-1	vascular cell adhesion molecule 1
VEGF	vascular endothelial growth factor
VLDL	very-low-density lipoprotein

## Appendix A

**Table A1:** PubMed search strategy, screening stages, and selection counts.

Search Query, Number of Records Retrieved (N)	Full Text of the Query
#1 Sea buckthorn and complementary evidence *n* = 1842	(((“Hippophae rhamnoides”[tiab] OR “Hippophaë rhamnoides”[tiab] OR “Elaeagnus rhamnoides”[tiab] OR Hippophae[tiab] OR Hippophaë[tiab] OR “sea buckthorn”[tiab] OR “sea-buckthorn”[tiab] OR seabuckthorn[tiab]) AND (leaf[tiab] OR leaves[tiab] OR foliar[tiab] OR twig[tiab] OR twigs[tiab] OR bark[tiab] OR berry[tiab] OR berries[tiab] OR fruit[tiab] OR fruits[tiab] OR seed[tiab] OR seeds[tiab] OR oil[tiab] OR oils[tiab] OR juice[tiab] OR puree[tiab] OR tea[tiab] OR extract[tiab] OR extracts[tiab] OR extraction[tiab] OR flavonoid[tiab] OR flavonoids[tiab] OR flavonol[tiab] OR flavonols[tiab] OR phenol[tiab] OR phenols[tiab] OR phenolic[tiab] OR phenolics[tiab] OR polyphenol[tiab] OR polyphenols[tiab] OR triterpene[tiab] OR triterpenes[tiab] OR triterpenoid[tiab] OR triterpenoids[tiab] OR tannin[tiab] OR tannins[tiab] OR ellagitannin[tiab] OR ellagitannins[tiab] OR carotenoid[tiab] OR carotenoids[tiab] OR tocopherol[tiab] OR tocopherols[tiab] OR tocotrienol[tiab] OR tocotrienols[tiab] OR phytochemical[tiab] OR phytochemicals[tiab] OR constituent[tiab] OR constituents[tiab] OR cardiovascular[tiab] OR cardiac[tiab] OR myocardial[tiab] OR metabolic[tiab] OR metabolism[tiab] OR diabetes[tiab] OR diabetic[tiab] OR glucose[tiab] OR insulin[tiab] OR obesity[tiab] OR obese[tiab] OR adiposity[tiab] OR steatosis[tiab] OR lipid[tiab] OR lipids[tiab] OR cholesterol[tiab] OR triglyceride[tiab] OR triglycerides[tiab] OR hypertension[tiab] OR hypertensive[tiab] OR “blood pressure”[tiab] OR endothelium[tiab] OR endothelial[tiab] OR vascular[tiab] OR platelet[tiab] OR platelets[tiab] OR thrombosis[tiab] OR thrombotic[tiab] OR antioxidant[tiab] OR antioxidants[tiab] OR oxidative[tiab] OR inflammation[tiab] OR inflammatory[tiab] OR ischemia[tiab] OR ischemic[tiab] OR hypoxia[tiab] OR hypoxic[tiab] OR toxicity[tiab] OR toxicological[tiab] OR safety[tiab] OR postprandial[tiab] OR sympathetic[tiab])) OR (“natural products”[tiab] AND “cardiovascular diseases”[tiab] AND (Nrf2[tiab] OR “anti-inflammatory”[tiab] OR antioxidant[tiab] OR antioxidants[tiab])) OR (Nrf2[tiab] AND “heart failure”[tiab] AND cardioprotection[tiab]) OR ((flavolignan[tiab] OR flavolignans[tiab]) AND silymarin[tiab] AND Nrf2[tiab]) OR (“activation of eNOS”[tiab] AND (polyphenol[tiab] OR polyphenols[tiab] OR “polyphenol-rich”[tiab])) OR (“endothelial dysfunction”[tiab] AND “clinical implications”[tiab] AND cardiovascular[tiab]) OR (“oxidative stress”[tiab] AND (antioxidant[tiab] OR antioxidants[tiab]) AND “vascular inflammation”[tiab] AND cardiovascular[tiab]) OR ((triterpene[tiab] OR triterpenes[tiab] OR triterpenoid[tiab] OR triterpenoids[tiab]) AND “immunomodulatory properties”[tiab]) OR (“urolithin A”[tiab] AND (immunomodulatory[tiab] OR immunomodulation[tiab]) AND “metabolic diseases”[tiab]) OR ((ellagitannin[tiab] OR ellagitannins[tiab]) AND “therapeutic potential”[tiab]) OR (carotenodermia[tiab] AND (diet[tiab] OR dietary[tiab]))) AND (“2000/01/01”[dp]: “2026/07/01”[dp])
#2 Flavonols and related mechanisms *n* = 1732	(((isorhamnetin[tiab] OR quercetin[tiab] OR kaempferol[tiab]) AND (endothelium[tiab] OR endothelial[tiab] OR HUVEC[tiab] OR HUVECs[tiab] OR vascular[tiab] OR “blood vessel”[tiab] OR “blood vessels”[tiab] OR aorta[tiab] OR artery[tiab] OR arteries[tiab] OR plaque[tiab] OR plaques[tiab] OR atherosclerosis[tiab] OR atherosclerotic[tiab]) AND (eNOS[tiab] OR NADPH[tiab] OR Nrf2[tiab] OR apoptosis[tiab] OR oxLDL[tiab] OR “oxidized LDL”[tiab] OR caveolin[tiab] OR “angiotensin II”[tiab] OR adhesion[tiab] OR heterocellular[tiab] OR vasorelaxation[tiab])) OR (quercetin[tiab] AND “vascular endothelium”[tiab] AND inflammation[tiab] AND “lipid metabolism”[tiab]) OR (isorhamnetin[tiab] AND (cardiac[tiab] OR cardioprotective[tiab] OR cardioprotection[tiab] OR myocardial[tiab] OR atrial[tiab] OR ischemia[tiab] OR ischemic[tiab] OR reperfusion[tiab]) AND (injury[tiab] OR injuries[tiab] OR remodeling[tiab] OR remodelling[tiab] OR cardioprotective[tiab] OR cardioprotection[tiab] OR apoptosis[tiab] OR ENO1[tiab] OR stem[tiab] OR “stem cell”[tiab] OR “stem cells”[tiab])) OR (quercetin[tiab] AND (“blood pressure”[tiab] OR hypertension[tiab] OR hypertensive[tiab] OR “myocardial infarction”[tiab]) AND (trial[tiab] OR trials[tiab] OR supplementation[tiab] OR patient[tiab] OR patients[tiab] OR subject[tiab] OR subjects[tiab])) OR (kaempferol[tiab] AND (“cardiovascular abnormalities”[tiab] OR hypertension[tiab] OR hypertensive[tiab] OR “blood pressure”[tiab]) AND (“nitric oxide”[tiab] OR TNF[tiab] OR rat[tiab] OR rats[tiab])) OR ((isorhamnetin[tiab] OR quercetin[tiab]) AND (antiplatelet[tiab] OR anticoagulant[tiab] OR antithrombotic[tiab] OR “platelet aggregation”[tiab] OR “thrombin-induced”[tiab])) OR (quercetin[tiab] AND isorhamnetin[tiab] AND “glucose uptake”[tiab]) OR (kaempferol[tiab] AND Nrf2[tiab] AND (“HO-1”[tiab] OR “ICAM-1”[tiab] OR pyroptosis[tiab])) OR (isorhamnetin[tiab] AND “oxidative stress”[tiab] AND Nrf2[tiab] AND “target genes”[tiab]) OR (quercetin[tiab] AND “anti-inflammatory potential”[tiab]) OR ((quercetin[tiab] OR isorhamnetin[tiab]) AND (glucoside[tiab] OR glucosides[tiab] OR glycoside[tiab] OR glycosides[tiab]) AND (bioavailability[tiab] OR bioavailable[tiab] OR bioaccessibility[tiab] OR bioaccessible[tiab] OR hydrolyzed[tiab] OR hydrolysed[tiab] OR hydrolysis[tiab] OR “intestinal permeability”[tiab])) OR ((polyphenol[tiab] OR polyphenols[tiab]) AND “metabolic fate”[tiab] AND (intestine[tiab] OR intestinal[tiab] OR colon[tiab] OR microbiota[tiab])) OR ((urolithin[tiab] OR urolithins[tiab]) AND bacteria[tiab] AND metabolism[tiab])) AND (“2000/01/01”[dp]: “2026/07/01”[dp])
Combined PubMed results	Search #1: *n* = 1842; search #2: *n* = 1732; raw total: *n* = 3574; overlapping records removed: *n* = 35; unique records: *n* = 3539; supplementary Scopus search: *n* = 0 unique records.
Title screening	Screened: *n* = 3539; excluded: *n* = 2738; retained for abstract assessment: *n* = 801. Reasons: insufficient relevance to the review question; disease area outside the review scope; agriculture, ecology, plant genetics, breeding, food technology, or animal feeding without a direct health question; insufficient relevance after manual assessment of a borderline title.
Abstract assessment	Records with an abstract: *n* = 789; records without an abstract: *n* = 12; excluded in the sensitive abstract screen: *n* = 332; retained after the sensitive screen: *n* = 469. Reasons: insufficient direct relevance or additional evidence; excluded topic; redundant evidence; correction, protocol, or record without independent results.
Global comparison	Assessed: *n* = 469; excluded: *n* = 321; retained for detailed assessment: *n* = 148. Reason: a retained source provided stronger, more direct, or less redundant evidence on the same issue.
Detailed assessment	Assessed: *n* = 148; not retained: *n* = 63; included in the manuscript: *n* = 85. All included publications were retrieved through PubMed and were in English.
