# Supplementary material for: Sea Buckthorn in Cardiovascular and Metabolic Health: Evidence for the Role of the Leaves
Source: Nutrients. 2026 Sep 14;18(18):3009. doi: 10.3390/nu18183009 (PMC13610675; doi:10.3390/nu18183009)
Supplement: Supplementary file 1 [file nutrients-18-03009-s001.zip › nutrients-4518038-supplementary.pdf]

nutrients MDPI

Table S1. Reported composition of sea buckthorn leaves and leaf preparations.

Reference/DOI	Material/Basis	Representative Constituent(s) or Measure(s)	Reported Value(s)
Sytařová et al., 2020 [6] 10.1016/j.foodchem.2019.125784	Leaves, nine cultivars; FM	total polyphenols	1.88–3.72 g GAE/kg
		total flavonoids	14.40–49.58 mg RE/kg
		vitamin C	22.81–46.32 g/kg
		vitamin E	71.54–153.99 mg/kg
		chlorogenic acid	198.9–3906.3 mg/kg
		epigallocatechin	231.4–2189.5 mg/kg
		catechin	100.1–2030.0 mg/kg
		rutin	7.3–23.7 mg/kg
		ellagic acid	2.3–165.8 mg/kg
He et al., 2023 [16] 10.1002/fsn3.3155	Commercial leaf teas (n = 18); dry product	ellagic acid	1.57–5.87 mg/g
		quercitrin	0.17–1.84 mg/g
		isoquercitrin	0.21–0.88 mg/g
		rutin	0.03–0.87 mg/g
		narcissin	0.07–0.94 mg/g
	Commercial leaf teas (n = 18); dry product; detected in 6/18 products	catechin	0.62–3.38 mg/g
	Commercial leaf teas (n = 18); dry product; detected in 15/18 products	epicatechin	0.09–0.40 mg/g
	Commercial leaf teas (n = 18); dry product; detected in 16/18 products	kaempferol	0.032–0.116 mg/g
	Commercial leaf teas (n = 18); dry product; detected in 2/18 products	apigenin	0.002–0.003 mg/g
	Commercial leaf teas (n = 18); dry product; detected in 18/18 products	isorhamnetin-3-O-neohesperidoside	0.002–0.019 mg/g
Yuan et al., 2025 [17] 10.1002/fsn3.70013	Dried leaf powder, 26 accessions; moisture basis NR	total flavonoids	>2% in most accessions; >3.5% in S19 and S22
		total polyphenols	>2% in most accessions; >4% in S1, S8, and S21

		crude protein	>15% in most accessions, >23% in S1
		crude fiber	>9% in most accessions, >16% in S4 and S13
		crude fat	>3% in most accessions, >6% in S8
		polysaccharides	>4% in most accessions, >8% in S4, S5, S7, and S22
		Na	25.67–45.64 mg/100 g
		K	12.88–26.76 mg/100 g
		Ca	116.04–142.2 mg/100 g
		Mg	118.01–364.16 mg/100 g
		Fe	16.38–54.2 mg/100 g
		Mn	9.14–88.95 mg/100 g
		Cu	0.8–7 mg/100 g
		Zn	6.86–27.43 mg/100 g
		caffeine	>325 mg/100 g
		vitamin C	>80 mg/100 g
Criste et al., 2020 [19] 10.3390/molecules25051170	50% ethanol leaf extract, four cultivars; basis (a)	total polyphenols	41.60–48.12 mg GAE/g
		total flavonoids	31.53–36.58 mg QE/g
		quercitrin	151.5–732.8 mg/100 g
		quercetin-3-galactoside	28.37–91.86 mg/100 g
		myricetin	19.36–34.13 mg/100 g
		rutin	5.84–25.74 mg/100 g
		gallic acid	0.88–8.90 mg/100 g
		chlorogenic acid	2.62–6.24 mg/100 g
		kaempferol	1.50–3.11 mg/100 g
		quercetin	8.69 in one cultivar mg/100 g
Wang et al., 2022 [7] 10.3389/fnut.2022.890486	Naturally dried leaves; four batches; basis (b)	total polyphenols	103.35–109.14 mg GAE/g
		total flavonoids	24.57–34.83 mg rutin/g
	Green leaf tea; four batches; basis (b)	total polyphenols	90.39–103.55 mg GAE/g
		total flavonoids	26.66–33.34 mg rutin/g
		total polyphenols	49.86–55.74 mg GAE/g

	Black leaf tea; four batches; basis (b)	total flavonoids	13.35–16.87 mg rutin/g
	Leaves and processed leaf teas	phenolics and other nonvolatile metabolites	48 compounds identified by LC–MS; individual concentrations NR
Ma et al., 2019 [23] 10.1016/j.foodchem.2018.08.006	Leaf infusion, 1 g leaves/100 mL water	total flavonol glycosides	9.73–11.72 mg/100 mL
		total ellagitannins	9.18–20.10 mg/100 mL
		total phenolics	39.26–64.34 mg/100 mL
	Fresh leaves and infusion residues; DW	total flavonol glycosides	Fresh leaves: 13.1–14.1 mg/g; residues: 5.8–7.4 mg/g
		total ellagitannins	Fresh leaves: about 30 mg/g; residues: 4.0–15.2 mg/g
	Leaf infusion, 1 g leaves/100 mL water	isorhamnetin-3-O-glucoside-7-O-rhamnoside	1.44–2.02 mg/100 mL
		isorhamnetin-3-O-rutinoside	0.61–1.51 mg/100 mL
		kaempferol-3-O-hexoside-7-O-rhamnoside	0.28–1.58 mg/100 mL
		pedunculagin	0.60–1.75 mg/100 mL
		stachyurin	2.74–9.07 mg/100 mL
		hippophaenin C	0.28–1.51 mg/100 mL
		casuarinin	3.14–6.73 mg/100 mL
		hippophaenin B	0.30–1.46 mg/100 mL
		casuarictin	0.84–2.62 mg/100 mL
Kubczak et al., 2022 [24] 10.1038/s41598-022-05104-2	50% ethanol leaf extract; dry extract	total phenolic compounds	269.49 mg/g
		total flavonoids	23.7 mg/g
		total catechins	9.3 mg/g
		procyanidin B2	13.933 ± 1.276 mg/g
		rosmarinic acid	11.642 ± 1.065 mg/g
		rutin	7.791 ± 0.731 mg/g
		ellagic acid	4.444 ± 0.416 mg/g
	50% ethanol leaf extract; dry extract (c)	total amino acids	0.62% in Results; 6.18 mg/g in Table 2; sum of listed amino acids: 61.79 mg/g
	50% ethanol leaf extract; dry extract	vitamin B1	3.0 ± 0.19 mg/g
		vitamin B2	4.0 ± 0.21 mg/g
		vitamin B3	3.0 ± 0.14 mg/g

		vitamin B5	2.8 ± 0.09 mg/g
		vitamin B6	5.4 ± 0.22 mg/g
		folic acid	0.8 ± 0.07 mg/g
		α-tocopherol	38.2 ± 0.22 μg/g
		β-tocopherol	10.6 ± 0.18 μg/g
		γ-tocopherol	8.30 ± 0.05 μg/g
Suvanto et al., 2018 [25] 10.1021/acs.jafc.7b04834	Dried leaves; DW; pedunculagin equivalents	total of 10 ellagitannins	42.5–109.1 mg/g
	Dried leaves; share of total ellagitannins	casuarinin	11.6–24.8 %
		stachyurin	7.7–24.8 %
		hippophaenin C	12.7–22.7 %
		combined share of casuarinin, stachyurin, and hippophaenin C	39.6–62.6 %
	Dried leaves; DW; mean ± SD	total ellagitannins: cultivar R	77.9 ± 3.1 mg/g
		total ellagitannins: cultivar K	68.5 ± 3.3 mg/g
		total ellagitannins: cultivar RUXRA	69.3 ± 3.8 mg/g
		total ellagitannins: male	74.3 ± 3.0 mg/g
		total ellagitannins: female	68.9 ± 2.8 mg/g
Zhao et al., 2024 [22] 10.3390/antiox13070763	Purified leaf flavonoid fraction; dry fraction	total quantified flavonoids	64.35 ± 0.79 mg/g
	Purified leaf flavonoid fraction; share of total flavonoids	quercetin and isorhamnetin glycosides	78.13 %
		kaempferol glycosides	17.39 %
		isorhamnetin-3-O-glucoside-7-O-rhamnoside	17.74 %
		kaempferol-3-O-sophoroside-7-O-rhamnoside	13.38 %
		rutin	13.17 %
		quercetin-3-O-galactoside	12.74 %
	Purified leaf flavonoid fraction	flavonoid profile	57 compounds identified; most individual absolute concentrations NR
Pop et al., 2014 [28] 10.1016/j.foodchem.2013.09.083	Leaves, six cultivars; DW	total carotenoids	3.5–4.2 mg/100 g
	Leaves, six cultivars; DW; ranges of cultivar means	neoxanthin	0.5–0.6 mg/100 g
		violaxanthin	0.5–0.6 mg/100 g

		lutein	0.8–1.1 mg/100 g
		zeaxanthin	0.5–0.6 mg/100 g where detected; ND in 3/6 cultivars
		β-carotene	0.7–0.9 mg/100 g
		cis β-carotene (peak 10)	0.5–0.6 mg/100 g where detected; ND in 1/6 cultivars
		cis β-carotene (peak 11)	0.5–0.6 mg/100 g
Kim et al., 2011 [21] 10.1021/jf103130a	Methanolic extract of leaves; mean ± SD, n = 3	total phenolic compounds	190 ± 3 mg GAE/g
	n-hexane fraction of leaves; mean ± SD, n = 3	total phenolic compounds	48 ± 6 mg GAE/g
	n-butanol fraction of leaves; mean ± SD, n = 3	total phenolic compounds	477 ± 7 mg GAE/g
	aqueous fraction of leaves; mean ± SD, n = 3	total phenolic compounds	173 ± 12 mg GAE/g
	Leaf extract; isolated compounds; native leaf content NR	kaempferol-3-O-β-D-(6''-O-coumaryl) glycoside	6.8 ± 0.3 mg/g extract
		1-feruloyl-β-D-glucopyranoside; isorhamnetin-3-O-glucoside; quercetin 3-O-β-D-glucopyranoside; quercetin 3-O-β-D-glucopyranosyl-7-O-α-L-rhamnopyranoside; isorhamnetin-3-O-rutinoside	1.8–6.1 mg/g extract across these five compounds; individual values NR
Sokolova et al., 2025 [26] 10.1080/14786419.2025.2534685	Leaf fractions; native leaf content NR	casuarinin	Fractions enriched predominantly in casuarinin; amounts NR
		casuarinin; casuarictin	Fractions enriched in both compounds; amounts NR
	Isolated casuarictin	casuarictin purity	94 %
Raclariu-Manolică & Socaciu, 2023 [31] 10.3390/foods12244493	Leaves; relative MS signals	luteolin-5-glucoside	NR
		arginine	NR
		isorhamnetin-3-rutinoside	NR
		serotonin	NR
		tocopherol	NR
Pariyani et al., 2020 [20] 10.1111/1750-3841.15025	Lyophilized leaves; two cultivars, locations, and seasons; untargeted NMR	phytosterols; linoleic and α-linolenic acids; threonine; alanine; glutamic acid; GABA; fructose; α-glucose; β-glucose; sucrose; myo-inositol; gluconic acid; ellagitannins; flavonol glycosides; gallic acid; ellagic acid; acetate; succinate; choline; trigonelline	20 identities; absolute concentrations NR

		polyphenols; fatty acids	Higher relative NMR signals in early harvest
		carbohydrates; sugars	Higher relative NMR signals in later harvest
		sugars	Higher relative NMR signals in the north
		ellagitannins	Higher relative NMR signals in the south
		fatty acids; carbohydrates; sugars	Higher relative NMR signals in Terhi
		ellagitannins; flavonol glycosides	Higher relative NMR signals in Tytti
Kwon et al., 2017 [8] 10.3390/nu9060569	80% aqueous ethanol leaf extract and its flavonoid-glycoside fraction; isolated compounds; concentrations in the administered preparations NR (d)	isorhamnetin 3-glucoside	4.7 mg isolated; isolation recovery, not concentration in the extract
		quercetin 3-glucoside	5.3 mg isolated; isolation recovery, not concentration in the extract
Skalski et al., 2023 [69] 10.1016/j.biopha.2023.114594	80% methanol leaf extract followed by defatting, butanol partitioning, and freeze-drying; dry extract (e)	ellagitannins, ellagic acid, and ellagic acid glycosides	259.6 ± 3.1 mg GAE/g
		casuarinin; hippophaenin B; casuarictin; stachyurin; strictinin; corresponding isomers	Included in the preceding class total; individual values NR
		glycosides of isorhamnetin, quercetin, and kaempferol	74.7 ± 0.7 mg RE/g
		isorhamnetin 3-O-glucoside-7-O-rhamnoside; rutin; kaempferol 3-O-coumaroylhexoside; corresponding isomers	Included in the preceding class total; individual values NR
		catechin and proanthocyanidins	7.2 ± 0.2 mg epicatechin equivalents/g
		triterpenoid saponins; triterpenoid acids; hydroxycinnamate-acylated triterpenoid acids	Identified; quantitative values NR
Pap et al., 2021 [81] 10.1016/j.fct.2021.112284	Aqueous leaf extract obtained by maceration; raw-material wet basis (f)	total phenolic content	2119 ± 31 mg GAE/100 g
		condensed tannins	218 ± 6 mg CE/100 g
	Aqueous leaf extract obtained by pressurized hot-water extraction; raw-material wet basis (f)	total phenolic content	1402 ± 8 mg GAE/100 g
		condensed tannins	86 ± 5 mg CE/100 g
	Freeze-dried aqueous leaf extract obtained by maceration (f)	quercetin	21.10 ± 0.50 mg/g
		3,4,5-trihydroxybenzoic acid	13.90 ± 0.30 mg/g
		ellagic acid	1.50 ± 0.60 mg/g
		proanthocyanidins	9287 ± 711 mg/100 g
		mean degree of polymerization	2.80 ± 0.07
		procyanidins	58.7% of proanthocyanidins

prodelphinidins	41.3% of proanthocyanidins
ellagitannins	3148 ± 35 mg/100 g

Abbreviations: CE, catechin equivalents; DW, dry weight; FM, fresh matter; FW, fresh weight; GABA, γ-aminobutyric acid; GAE, gallic acid equivalents; LC–MS, liquid chromatography–mass spectrometry; MS, mass spectrometry; ND, not detected; NMR, nuclear magnetic resonance; NR, not reported; QE, quercetin equivalents; RE, rutin equivalents; SD, standard deviation. Note: Table S1 summarizes nutritional and phytochemical data reported for sea buckthorn leaves and leaf preparations in the studies cited in this review. Values are retained in the terminology, units, and analytical bases of the original articles to preserve distinctions among plant materials, extracts, fractions, teas, and analytical methods. Values for extracts, fractions, teas, and infusions do not represent native leaf content. Where the composition of a cited experimental preparation was not quantified in the source, this is indicated as NR. For Pariyani et al., higher or lower indicates relative NMR signal abundance, not concentration. Source notes: Sytařová et al., total flavonoids and the units for vitamin E and ellagic acid follow Tables 1 and 3; the abstract gives 49.44 mg RE/kg instead of 49.58 mg RE/kg. Yuan et al., Figure 2 was used to distinguish total polyphenols from total flavonoids. (a) Criste et al.: Methods give totals per g of extract, but Results label the basis as FW. (b) Wang et al.: total polyphenol ranges follow the Results because decimal points are missing from Table 2; total flavonoid ranges follow Table 2 because the Results paragraph differs slightly. (c) Kubczak et al.: Results give 0.62%, Table 2 gives 6.18 mg/g, and the listed amino acids sum to 61.79 mg/g; all values are retained because the source is internally inconsistent. (d) Kwon et al.: The reported amounts are recoveries of compounds isolated during chromatographic characterization and do not represent their concentrations in the administered leaf extract or flavonoid-glycoside fraction. Quantitative concentrations in these preparations were not reported. (e) Skalski et al.: Phenolic compounds were determined semi-quantitatively. Class totals were expressed using gallic acid for ellagitannins and ellagic acid derivatives, rutin for flavonoids, and epicatechin for catechin and proanthocyanidins. Individual compounds identified within these classes were not quantified separately. Only the leaf-extract data were included. (f) Pap et al.: Results for maceration and pressurized hot-water extraction were retained separately. Total phenolics and condensed tannins are expressed relative to the raw material on a wet basis, whereas individual compounds, proanthocyanidins, and ellagitannins refer to the freeze-dried extract obtained by maceration.
